# A new *Stamnodes* from the southwestern United States (Lepidoptera, Geometridae, Larentiinae)

**DOI:** 10.3897/zookeys.923.48290

**Published:** 2020-04-01

**Authors:** Tanner A. Matson, David L. Wagner

**Affiliations:** 1 Department of Ecology and Evolutionary Biology, University of Connecticut, Storrs, Connecticut 06269–3043, USA University of Connecticut Storrs United States of America

**Keywords:** COI, DNA barcodes, Lamiaceae, *
Salvia
*, shrubby blue sage, Stamnodini

## Abstract

*Stamnodes fergusoni***sp. nov.** occurs from extreme southeastern Arizona through southern New Mexico east into western Texas, USA. Identity of the new species can be reliably determined by external features, genitalic characters, and COI haplotypes. Larvae are believed to be specialists on *Salvia pinguifolia* and *S. ballotiflora*. The adult and larval stages and male and female genitalia are illustrated, available DNA barcode data that support the recognition of the new *Stamnodes* are reviewed, and its life history briefly characterized.

## Introduction

*Stamnodes* Guenée is one of the most handsome, species-rich, and taxonomically problematic genera of North American Geometridae. Several species remain undescribed and much disagreement surrounds the validity of many recognized species and their current synonymies. The description of *Stamnodes fergusoni* is long overdue; for nearly three decades this species has been known by moth collectors and photographers of west Texas as undescribed (Knudson and Bordelon 2002, Ed Knudson pers. comm. (recently deceased)) and is identified in on-line identification resources (e.g., iNaturalist and BugGuide) as a new taxon. Douglas Ferguson, the former curator of the Geometridae at the USDA-United States Natural History Museum (USNM), had initiated a manuscript describing the species, but died before he could complete the work.

Here we describe *Stamnodes fergusoni* from southwestern Texas’s hill country, westward through New Mexico into extreme southeastern Arizona. The new taxon is believed to be a specialist on woody mints, feeding on *Salvia pinguifolia* (Lamiaceae) in New Mexico and Arizona and presumably on *Salvia ballotiflora* (Lamiaceae) over the core of its range in southwest Texas. We describe and illustrate the larval and adult stages of the new species, illustrate the male and female genitalia, give diagnostic differences that separate this taxon from two phenotypically similar North American congeners: *Stamnodes fervefactaria* (Grote, 1881) and *Stamnodes deceptiva* (Barnes and McDunnough, 1917), and provide a brief account of its biology and distribution.

## Materials and methods

Adults were obtained by light trapping with UV and mercury-vapor lights. Larvae were collected from *Salvia pinguifolia* (Lamiaceae) near Carrizozo, New Mexico and Warren, Arizona. The holotype was collected by Jim Vargo in Val Verde Co., TX. The adult description of *S. fergusoni* is based on 74 pinned specimens and 15 additional photographic records; the larval description is based on five larvae (DLW Lot: 2018J32, 2018J35, and 2018J133). 139 genitalic slides of *Stamnodes* were examined: 73 examined from the National Museum of Natural History (USNM), 63 on loan from the Canadian National Collection (CNC), and one slide prepared by TAM for this study. Two slides of *S. fergusoni*, four slides of *S. deceptiva*, and four slides of *S. fervefactaria* were examined. Doug Ferguson’s unfinished manuscript describing the new moth was made available by Dr. Alma Solis. In both the *Diagnosis* and *Description*, we sometimes reproduce Doug’s original text for genitalic characters; Ferguson’s text was both concise and accurate and we could not do better.

Over the course of this study we examined the Nearctic *Stamnodes* holdings (including primary types) of the American Museum of Natural History (**AMNH**) (New York City, NY), Harvard University (**MCZ**) (Cambridge, MA), McGuire Center for Lepidoptera and Biodiversity (**MGCL**) (University of Florida, Gainesville, FL); National Museum of Natural History (**NMNH**) (Washington DC); University of Connecticut (**UCMS**) (Storrs, CT); as well as specimens gifted to us by Edward C. Knudson. We had access to 457 Stamnodini COI barcode submissions: for each of these, we examined the associated voucher specimen or image (when available). 378 of the 457 COI barcodes were used (failed sequences excluded) to generate a neighbor-joining tree, using the default Kimura-2P model in the Barcode of Life Project (BOLD) (http://www.boldsystems.org) (Ratnasingham and Hebert 2007). DNA extraction, PCR amplification, and COI barcode sequencing were performed at the Canadian Centre for DNA Barcoding (Centre for Biodiversity Genomics–University of Guelph) using their standard Sanger sequencing protocols (Wilson 2012). SimpleMappr (www.simplemappr.net) was used to generate the geographic distribution point map (Shorthouse 2010). Type material has been deposited in the USNM, AMNH, UCMS, TAMUIC, and KSU-MEPAR collections.

## Taxonomy

### Stamnodes fergusoni sp. nov.

Taxon classificationAnimaliaLepidopteraGeometridae

BE43FD77-6977-51DB-B76A-67DF5EFCE10A

http://zoobank.org/ABACB3A5-E374-4ABE-863F-0C46F73E8A61

**Figures 1–3.**
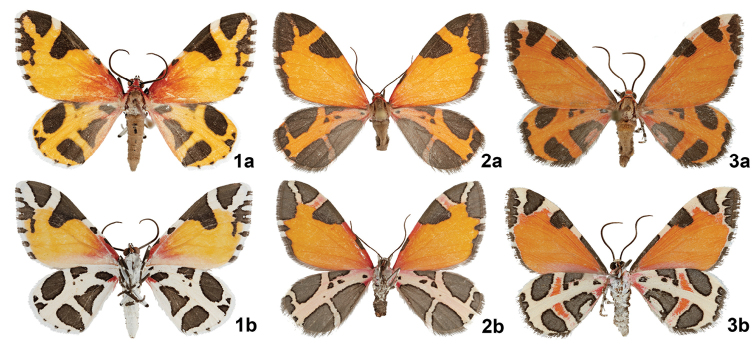
Adult *Stamnodes*. **1***Stamnodes fergusoni* [HOLOTYPE], TX: Val Verde Co., Ranch Road 189 (30.1823°, -100.06°), 19 September 2018, J. Vargo coll., **a** dorsal view, **b** ventral view; **2***Stamnodes fervefactaria*, Colorado: El Paso Co., ssw of Colorado Springs Hwy. #115 at mkr. #30.2 Los Pinos housing entry [38.5797, -104.9308], 6480ft, 14 August 2004, Chuck Harp coll., BOLD Process ID: LNAUW1876-17, Museum ID: USNM ENT 01343267, **a** dorsal view, **b** ventral view; **3***Stamnodes deceptiva*, AZ: Cochise Co., Ash Canyon Rd Huachuca Mts., 5100ft, 3 August 1999, Douglas C. Ferguson coll., BOLD Process ID: LNAUS1646-13, Museum ID: USNM ENT 00808502 **a** dorsal view, **b** ventral view.

**Figures 4–6.**
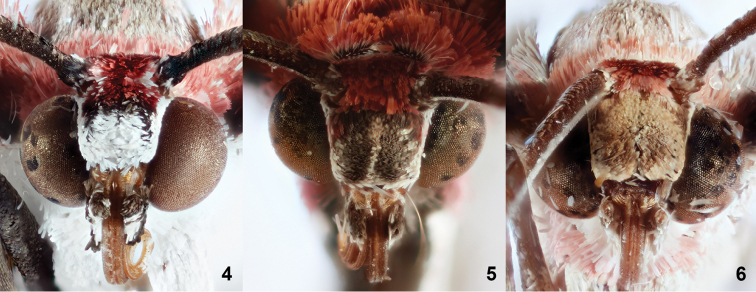
Frontal view of head for *Stamnodes fergusoni* and related species. **4***Stamnodes fergusoni*; **5***Stamnodes fervefactaria*; **6***Stamnodes deceptiva*.

**Figures 7–9.**
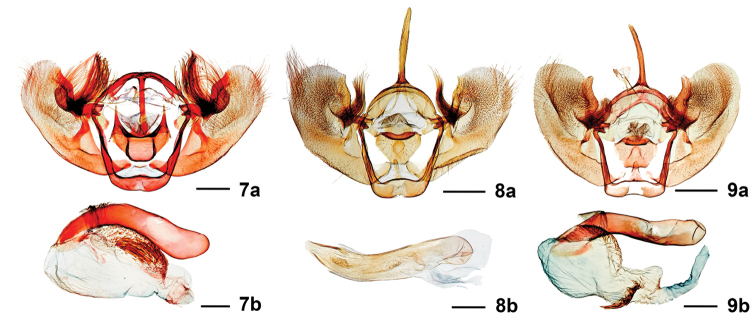
Male genitalia. **7***Stamnodes fergusoni* (USNM58902), **a** genital capsule, **b** aedeagus with everted vesica; **8***Stamnodes fervefactaria* (USNM55242), **a** genital capsule, **b** aedeagus; **9***Stamnodes deceptiva* (USNM58903), **a** genital capsule, **b** aedeagus with everted vesica. Scale bars: 0.85 mm (**9a**), 0.25 mm (**9b**).

**Figures 10–12.**
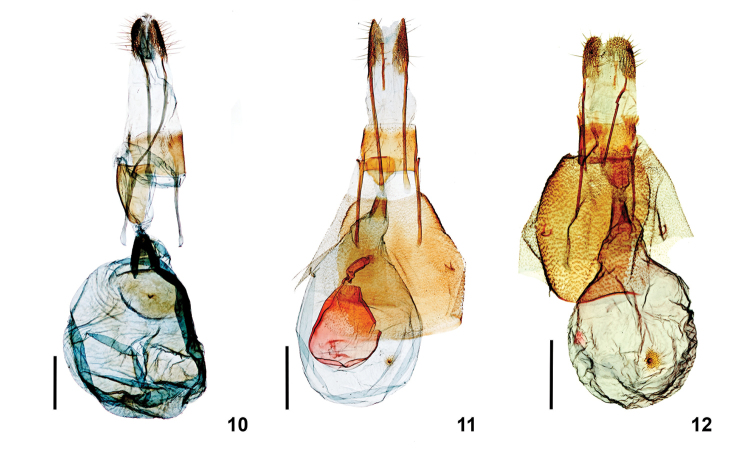
Female genitalia. **10***Stamnodes fergusoni* (TAM-2019-001); **11***Stamnodes fervefactaria* (USNM55243); **12***Stamnodes deceptiva* (USNM59079). Scale bar: 1 mm.

**Figure 13–14.**
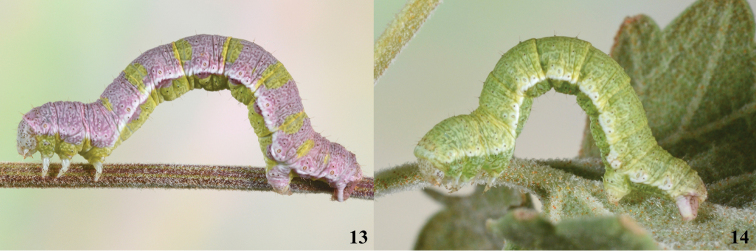
Larvae of *Stamnodes fergusoni* on *Salvia pinguifolia*; NM: Lincoln Co., Carrizozo lava flow, DLW Lot: 2018J32, COI Barcode DLW-001425. **13** last instar; **14** penultimate instar.

**Figure 15.**
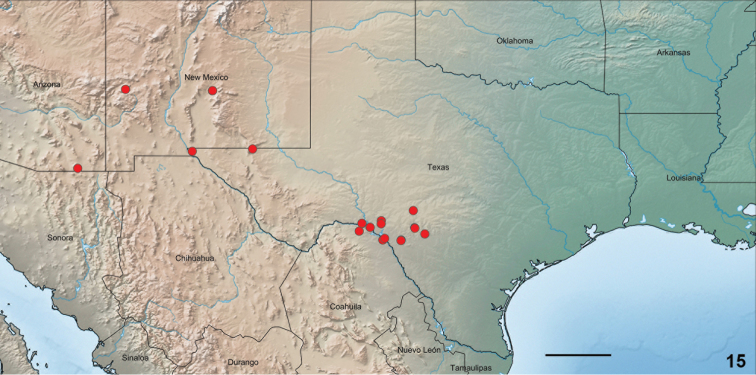
Distribution of *Stamnodes fergusoni* (*N* = 90, red). Single dots may represent > 1 individual. Scale bar: 200 km.

Figures 1 , 4 , 7 , 10 , 13 , 14 , 15

#### Diagnosis.

*Stamnodes fergusoni* can be immediately separated from most *Stamnodes* by its orange ground color and pattern of lead-gray patches across the forewing and hindwing. North of Mexico, *Stamnodes fergusoni* may only be confused with *Stamnodes deceptiva* or *Stamnodes fervefactaria*, neither of these species occur in southwest Texas. Although superficially close (and sometimes confused in collections), *S. fergusoni* can be quickly distinguished from the other two species. *Stamnodes fergusoni* can be reliably determined by the bright, white reticulate pattern of the underside of the hindwing and costal area of the forewing (Fig. 1b). In the other two species, the reticulate pattern of fore- and hindwing is cream to dull orange in color (Figs 2b, 3b). The frons of *S. fergusoni* is immediately diagnostic: the dorsal scarlet scales and ventral white scales are separated by a band of black scales (Fig. 4). *Stamnodes fergusoni* also differs from *S. deceptiva* and *S. fervefactaria* by its slightly larger size, lighter orange ground color, brighter scarlet scales at forewing base, and mostly scarlet tegula (Fig. 1).

Male genitalia (Fig. 7) are generally similar to those of *S. deceptiva* except for the following differences: (1) The costal sclerite is fully integrated into the valve and lacks the protruding thumb-like free end of *S. deceptiva*. (2) A prominent hair tuft arising from the low tubercle on the inner face of valve is approximately as long as the uncus or as long as the distance from its place of attachment to the end of the valve in *S. fergusoni*, but only half that length in *S. deceptiva*. (3) The juxta has a posterolateral pair of long, acuminate-conical processes that are lacking in *S. deceptiva*. (4) The vesica has ca. 30 large cornuti and many small cornuti clustered in one large group in *S. fergusoni*; there are two or three large and three or four small cornuti in one group, and many very small ones in another widely separate group in *S. deceptiva*. The male genitalia of *S. fervefactaria* are most similar to those of *S. deceptiva* except that the costal sclerite of the valve does not have a thumb-like free end and is nearly straight, not S-shaped as in *S. deceptiva*; the hair tuft on the valve reaches the end of the costal sclerite (but only half that distance in *S. deceptiva*); and the vesica has only small, poorly developed cornuti (Figs 8, 9). Female genitalia (Fig. 10) are similar to those of *S. deceptiva* and *S. fervefactaria* except for the following differences: (1) The bursa copulatrix has two widely separated signa, the one nearer to the ductus bursae is centered in a large sclerotized area and bears a single thorn-like process inwardly (on interior surface of bursa copulatrix); the other signa lacks a process; *Stamnodes deceptiva* has only a single nipple-like signum. (2) The ostium bursae is marked by a relatively large, strongly sclerotized, elongate, subtriangular plate (rounded anteriorly, slightly emarginate posteriorly). (3) Overall, the genitalia are ca. 1.5 × larger than those of *S. deceptiva* and *S. fervefactaria* (Figs 11, 12). The small sclerotized regions forming the bases of the signa of all species are usually depressed externally and convex internally, with or without an inner process. The signa of *S. fervefactaria* are reduced.

#### Description.

***Adult male.*** Forewing length: 14–16mm (Fig. 1) (*N* = 74). **Head.** Vertex scarlet, sometimes with scattered snow-white scales near base of antenna and collar; frons with dorsal scarlet and ventral white scales separated by thin transverse band of black scales; laterad black scales continued ventrad into white scaling; white scales along dorsal margin of eye. Labial palpus subequal to diameter of eye, short and slightly porrect, with exceedingly long black scales (especially near base) and sparse white intersegmental scales. Antenna filiform, laterally compressed, 0.5 length of forewing; scape mostly black, typically with small dorsal patch of white scales and several scattered white basal scales; flagellum fuscous with dorsal scales only, lateral and ventral sides bearing abundant microsetae. Collar scarlet with inconspicuous posterior white scales. Lateral patches of gray piliform scales between vertex and collar. **Thorax.** Mesoscutum predominantly light gray dorsally, generally trending from anterior dark gray (except white under patagium) to posterior cream-white, several individuals showing modest pink tone; ventrally bright white. Patagium scarlet; tegula mostly scarlet, basally black, and transitioning to pale, pink piliform scales posteriorly. Legs banded with fuscous and white; tibial spur formula 0–2–4. Coxae snow white. **Forewing.** Scarlet near base, diffusing to light orange near antemedial area (orange ground color paler than that of *S. deceptiva* and *S. fervefactaria*). Costal margin with several lead-gray patches; two small patches near base and antemedial area hugging costal margin (same patches continuous in *S. deceptiva*, and either singular or missing in *S. fervefactaria*); large trigonate to subquadranglar patch near postmedial area terminating near M_3_; apical patch more or less trigonate and highly variable, extending along outer margin before terminating prior to tornus, often divided by lobe or ray of orange ground color that extends toward apex. Fringe checkered, alternating white and lead-gray. Underside patterned like upperside. Scarlet base more vibrant on costa; costal and apical areas between lead-gray patches given much more to white than on upperside; checkered fringe pattern often extending into terminal area especially near apex. **Hindwing.** Reticulate light-orange ground color between variable patches and spots of lead-gray (more dissected than *S. fervefactaria*). Inner margin basally scarlet, quickly transitioning to admixture of fuscous, light orange, and scarlet. Approximately five ill-defined lead-gray patches (clockwise from wing base): small, basal oblong patch; small, often broken, costal, triangular antemedial patch; larger, ovate postmedial patch; ovate patch at anal angle, subequal to previous; and large, irregularly triangular patch that runs along inner margin. Often with irregular lead-gray subterminal line that expands into subapical patch, connecting dark areas in fringe. Fringe checkered, lead-gray scaling reduced to almost absent in some specimens. Underside like upperside, except light-orange ground color replaced by bright white, lead-gray patches finely outlined by dark gray, basal lead-gray patch often with small scarlet spot basad. **Abdomen.** Dorsum fuscous, venter pale gray; small black spiracular spots. **Male genitalia** (Fig. 7) (*N* = 2) Uncus long and narrow, tapering slightly at apex. Valva broadly ovate, ca. 2 × longer than wide, outwardly convex at middle of costal margin; broadly rounded at apex; prominent hair tuft arising from low tubercle on inner face of valve approximately as long as uncus or as long as distance from its place of attachment to end of valve. Juxta U-shaped, with posterolateral pair of long, acuminate-conical processes. Aedeagus cylindrical, exceeding length of valva; base broadly rounded; apex with broad concave aperture; vesica with ca. 30 large spinose cornuti surrounded by many smaller spinose cornuti in dense cluster.

***Female.*** Forewing length: 16–17mm (*N* = 9). Outwardly undifferentiated from male. **Female genitalia** (Fig. 10) (*N* = 1). Papillae anales broadly pointed and unfused posteriorly. Posterior apophysis fused with papillae anales anteriorly, long and extending to intersegment of A9 of A10; anterior apophysis one-half the length of posterior apophysis. Ostium bursae with large, ventral, strongly sclerotized, elongate, subtriangular plate (rounded anteriorly, slightly emarginate posteriorly). Ductus bursae short, sclerotized laterally, subtriangular and opening broadly into corpus bursae. Corpus bursae spheroid with two widely separated signa; signum nearer ductus bursae centered in large sclerotized area and bearing a single thorn-like process directed inward on interior surface; anterior signum without such a process and with rugose areole.

#### Description of living final instar

(Figs 13, 14). Coloration variable, dorsum ranging from green to lavender or mixture of both; venter yellow-green to lime-green, darker than dorsum in green forms. In cross section, venter shallowly hemispherical below white lateral stripe, appearing somewhat flattened. Upper side of white subspiracular stripe diffuse, enveloping spiracles; ventral margin more defined. Primary setae, sharp, relatively long, roughly 2.5-3 × height of spiracle, many borne from black pinaculum positioned atop minute wart. Spiracles tan to pale orange with darker peritreme. Head with circular freckles mostly clustered into tight groups over each lobe and in each corner of frons; primary setae from black pinacula; stemma 3 ca. 1.5 × stemmata 2 and 6.

#### Description of preserved penultimate instar.

Integument roughened and transversely furrowed. Extensively peppered with minute melanized patches which are most visible over each lobe of head and posterior abdominal segments (especially in cleared preparations). Prothoracic spiracle subequal to those on A1-A6 (~0.12 mm high); spiracle on A7 and A8 (~0.16 mm high). Planta of A6 with 20 or 21 secondary setae below level of SV seta, bearing 25 or 26 crochets mostly of two alternating lengths.

#### Type material examined.

***Holotype male***, TX: Val Verde Co., Ranch Road 189 (30.1823°, -100.06°), Elev. 1676’, 19 September 2018, Jim Vargo coll. dry pinned (USNM) (Fig. 1).

***Paratype adults*.** (52♂, 18♀): NM: Lincoln Co., Valley of Fires Recreation Area (33.67977°, -105.92708°), 11 August 2014, Black light trap, Leg. J. Luig & J. Metlevski, Voucher Codes: (363203, 363204), (2♂) (KSU-MEPAR); NM: Lincoln Co., Valley of Fires Recreation Area (33.681795°, -105.923486°), 4–5 September 2015, At light, Leg. J. Metlevski, Voucher Codes: (363197, 363198), (2♂) (KSU-MEPAR); NM: Lincoln Co., Valley of Fires Recreation Area (33.678049°, -105.927135°), 4 September 2015, Mercury vapor light, Leg. J. Metlevski, Voucher Codes: (363193–363196), (4♀) (KSU-MEPAR, TAMUIC); NM: Lincoln Co., Valley of Fires Recreation Area (33.678167°, -105.927492°), 4 September 2015, Black light trap, Leg. J. Metlevski, Voucher Codes: (363199–363202, 367370–367375), (9♂, 1♀) (TAMUIC, USNM, AMNH); TX: Uvalde Co., Concan, 9 November 2015, Ed Knudson coll., (1♂)(UCMS); TX: Uvalde Co., Concan, 28 September 2013 – 1 October 2013, Ed Knudson & Charles Bordelon colls., (1♂, 1♀) (UCMS); TX: Edwards Co., 1.2 km NW Camp Wood [29.6822°, -99.9711°], 13 October 2017, Ann Hendrickson coll., (3♂) (UCMS); TX: Val Verde Co., Seminole Canyon State Park, 20 October 1985, John Rawlins coll., BOLD Process IDs: LNAUV1793-17, LNAUV450-16, Museum IDs: USNM ENT 01276376, 01237649, Genitalic Slide DCF1651, (1♂, 1♀) (USNM); TX: Val Verde Co., Ranch Road 189 (30.1823°, -100.06°), 19 September 2018, Jim Vargo coll. (1♂, 1♀)(USNM); TX: Val Verde Co., (29.696°, -101.324°), 25 October 2014, Jim Vargo coll. (1♀)(TAMUIC); TX: Val Verde Co., Del Rio, 4 October 1994, Ed Knudson coll., (25♂, 7♀)(MGCL); TX: Uvalde Co., ConCan, 15 October 2001, G. Muise coll., (2♂, 1♀)(MGCL); TX: Uvalde Co., ConCan, 19 October 2000, Ed Knudson coll., (1♂)(MGCL); TX: El Paso Co., Franklin Mts., 17 September 1993, Ed Knudson coll., (3♂)(MGCL); TX: Val Verde Co., Dolan Falls Devil’s River, 3–10 October 1994, J. Gillaspy coll., (1♀)(MGCL); TX: Kinney Co., Bracketville, 5 October 1994, Ed Knudson coll., (1♂)(MGCL).

#### Other material examined.

***Adults.*** TX: (County Unknown), Langley [Langtry], 25 October 1945, (collector unknown), BOLD Process ID: LNAUV1794-17, Museum ID: USNM ENT 01276377, Genitalic Slide USNM58902, (1♂) (USNM); TX: Val Verde Co., Seminole Canyon State Park, 20 October 1985, John Rawlins coll., (damaged by museum pests) (1♂) (USNM); TX: Culberson Co., Guadalupe Mountains National Park north McKittrick Canyon, 28 March 1968, Roy O. Kendall & C. A. Kendall coll., (1♂) (MGCL); NM: Catron Co., Gila National Forest, Forest rd. 94 (33.7181°, -108.4683°), 20 August 2015, Elev. 8200’, Ron Parry coll. (from photograph). Additional internet photographic records (mapped in Figure 15) from iNaturalist (https://www.inaturalist.org/observations/): 18390285, 18339093, 9176399, 8832387, 8819801, 8813547, 8801663, 8795069, 8367680, 2360810. BugGuide observations (https://bugguide.net/node/view/): 1322692, 1493681, 1456212, 713503. ***Larvae.*** NM: Lincoln Co., Carrizozo lava flow, 16 September 2018, DLW Lot: 2018J32 & 2018J35, Tanner Matson & David Wagner coll., BOLD Process ID: WAGL1235-18, beaten from *Salvia pinguifolia* (*N* = 5) (UCMS); AZ: Cochise Co., Warren, 23 September 2018, DLW Lot: 2018J133, Tanner Matson, David Wagner, & Mimi Kamp coll., BOLD Process ID: WAGL1401-19, beaten from *Salvia pinguifolia* (*N* = 1) (UCMS).

#### Distribution.

(Fig. 15) Southwest Hill Country (Uvalde, Kinney, Edwards, and Val Verde counties); Franklin Mountains (Franklin County); and Guadalupe Mountains (Culberson County) of Texas (Knudson and Bordelon 2002, and this paper). Carrizozo Malpais lava flow (Lincoln County), New Mexico (where it is abdundant); also Gila National Forest (Catron County), New Mexico (https://southwesternmoths.com). Mule Mountains (Cochise County) of extreme southeastern Arizona. Range into Mexico remains unclarified; we have examined several collections from the north Coahuilan hill country near Ciudad Acuña.

#### Etymology.

The species is named in honor of the late Douglas C. Ferguson, a reigning and much respected authority on the geometrid fauna of North America for more than four decades. Dr. Ferguson’s notes, dissections, and collections were valuable assets that guided this effort. Dr. Ferguson was in the process of describing this species before his passing (Knudson and Bordelon 2002) (pers. comm. Charles Covell; Alma Solis).

#### Biology.

The larvae are believed to be specialists on woody *Salvia* (Lamiaceae). *Stamnodes fergusoni* larvae were collected and reared to maturity on *Salvia pinguifolia* (rock sage) from Carrizozo Malpais, New Mexico (BOLD Process ID: WAGL1235-18, BOLD Sample ID: DLW-001425). A middle instar larva was collected from *Salvia pinguifolia* in Warren, Arizona (BOLD Process ID: WAGL1401-19, BOLD Sample ID: DLW-001401). In the Southwestern Hill Country of Texas, the species is believed to feed on *Salvia ballotiflora* (shrubby blue sage) as this is the only woody *Salvia* at the localities where this species has been taken.

The larvae of *S. fergusoni* were first discovered on *Salvia pinguifolia* plants growing at the bottom of large gas bubbles that formed during the deposition of the Carrizozo Malpais lava field, west of Carrizozo, New Mexico. When the top of a bubble erodes away, the remaining volcanic pit acts as a catch basin for rain and dust, and over time comes to support diverse gardens in an otherwise black ultraxeric landscape. Larvae were found on plants growing within these bubbles, especially where overhanging lava shelves shield plants from full sun. Smaller, water-stressed plants growing in full sun yielded no *S. fergusoni* larvae. The purple pigments in the caterpillar appear to be derived from the flowers of its host (Fig. 13). In the laboratory, caterpillars were observed with their bodies deeply inserted into the calyx of individual flowers, feeding on reproductive tissues and callow seeds. The caterpillars of *S. deceptiva* and *S. fervefactaria* are so far unknown; we suspect they are using other Lamiaceae.

*Stamnodes fergusoni* has a single late-summer generation that appears to be tied to the flowering time of its preferred host. The moth is locally common in New Mexico and southwestern Hill Country of Texas with peak adult activity in September and October. Adults fly earlier (first weeks of September) in New Mexico. Several worn individuals were taken in March (2002) from the Guadalupe Mountains, by Roy Kendall. Knudson and Bordelon (2002) believed this collection represented overwintering adults. Mature larvae pupate in leaf litter or over soil. Over most of the species’ range, the pupa is believed to overwinter and remain in diapause until the following fall.

## Discussion

In June of this year, while working at the USNM, TAM had the pleasure to share desk space with Charles Covell. Charlie informed TAM about a new *Stamnodes* species collected by Ed Knudson in west Texas; the taxon was to be described by Doug Ferguson before his untimely passing. Charlie intended to publish Doug’s nearly complete version of this description with the type series Doug had received from Ed Knudson (now at MGCL). Coincidently, our manuscript treating the same moth, spurred by the discovery of the moth’s early stages, was nearing completion. With Dr. Covell’s and Dr. Alma Solis’s blessings, we utilize some of Doug’s text in both our diagnosis and genitalic description.

All larval collections are from *Salvia pinguifolia*, a plant that does not occur in southwestern Texas where *Stamnodes fergusoni* is locally common. The woody mint in this region is instead, *Salvia ballotiflora* (shrubby blue sage), which is seemingly the only candidate host for *Stamnodes fergusoni*. In conversations with DLW, Ed Knudson had mentioned that the moth was most commonly encountered in the Tamaulipan scrub associations of Val Verde County, Texas. DLW went to areas described by Knudson in November 2018, and found *S. ballotiflora* to be common through the county, although the third week of November was too late to find larvae and verify that shrubby blue sage is the primary host for this moth in south Texas.

We had access to barcode data for 457 *Stamnodes* and *Stamnoctenis* Warren specimens representing approximately 63 species-level taxa (bins), 378 of the 457 were used (failed sequences excluded). Surprisingly, barcode data placed *S. fergusoni* well outside of a sister relationship with *S. fervefactaria* and *S. deceptiva*, and offered no likely indication of its placement within *Stamnodes*. Uncorrected genetic divergence between the three taxa was greater than six percent for every relationship. Nuclear markers will be required to determine the placement of *S. fergusoni* within *Stamnodes*.

The most recent North American checklist of Lepidoptera (Ferguson, 1983), lists 34 valid species and numerous synonyms for *Stamnodes*. Much disagreement surrounds the validity of the currently recognized species and their synonymies. On-line identification sites (e.g., iNaturalist) and genetic databases (e.g., GenBank and Barcodes of Life) are riddled with misapplied names and generic-level (only) identifications. Institutional collections and published literature also reflect considerable taxonomic confusion. Our field collections of larvae for more than a twenty western *Stamnodes* and *Stamnoctenis* species, reveal considerable intraspecific variability in larval phenotypes, a matter further complicated by considerable host overlap across the genus. Such being the case, larvae and associated life history data will be of limited help in sorting out the species-level entities across the genus. Until a modern revisionary treatment employing nuclear markers, genitalic study, and larvae and their attendant life histories can be completed, databases, collections, and on-line resources treating North American Stamnodini will remain plagued with misidentifications and taxonomic uncertainty.

## Supplementary Material

XML Treatment for Stamnodes fergusoni

## Supplementary materials

Supplementary material 1 *Stamnodes fergusoni* collection records Data type species data https://binary.pensoft.net/file/396492This dataset is made available under the Open Database License (http://opendatacommons.org/licenses/odbl/1.0/). The Open Database License (ODbL) is a license agreement intended to allow users to freely share, modify, and use this Dataset while maintaining this same freedom for others, provided that the original source and author(s) are credited.Tanner A. Matson, David L. Wagner
